# Spectra Fusion of Mid-Infrared (MIR) and X-ray Fluorescence (XRF) Spectroscopy for Estimation of Selected Soil Fertility Attributes

**DOI:** 10.3390/s22093459

**Published:** 2022-05-01

**Authors:** Lalit M. Kandpal, Muhammad A. Munnaf, Cristina Cruz, Abdul M. Mouazen

**Affiliations:** 1 Department of Environment, Ghent University, Coupure Links 653, 9000 Gent, Belgium; lalitm85@gmail.com (L.M.K.); munnaf.mabdul@ugent.be (M.A.M.); 2 Centre for Ecology, Evolution and Environmental Changes (cE3c), Faculdade de Ciências da Universidade de Lisboa, Cidade Universitária, Bloco C2, 1749-016 Lisboa, Portugal; ccruz@fc.ul.pt

**Keywords:** precision agriculture (PA), multi-sensor, spectra fusion (SF), sequential orthogonalized partial least square (SOPLS), soil fertility

## Abstract

Previous works indicate that data fusion, compared to single data modelling can improve the assessment of soil attributes using spectroscopy. In this work, two different kinds of proximal soil sensing techniques i.e., mid-infrared (MIR) and X-ray fluorescence (XRF) spectroscopy were evaluated, for assessment of seven fertility attributes. These soil attributes include pH, organic carbon (OC), phosphorous (P), potassium (K), magnesium (Mg), calcium (Ca) and moisture contents (MC). Three kinds of spectra fusion (SF) (spectra concatenation) approaches of MIR and XRF spectra were compared, namely, spectra fusion-Partial least square (SF-PLS), spectra fusion-Sequential Orthogonalized Partial least square (SF-SOPLS) and spectra fusion-Variable Importance Projection-Sequential Orthogonalized Partial least square (SF-VIP-SOPLS). Furthermore, the performance of SF models was compared with the developed single sensor model (based on individual spectra of MIR and XRF). Compared with the results obtained from single sensor model, SF models showed improvement in the prediction performance for all studied attributes, except for OC, Mg, and K prediction. More specifically, the highest improvement was observed with SF-SOPLS model for pH [*R*^2^*p* = 0.90, root mean square error prediction (RMSEP) = 0.15, residual prediction deviation (RPD) = 3.30, and ratio of performance inter-quantile (RPIQ) = 3.59], successively followed by P (*R*^2^*p* = 0.91, RMSEP = 4.45 mg/100 g, RPD = 3.53, and RPIQ = 4.90), Ca (*R*^2^*p* = 0.92, RMSEP = 177.11 mg/100 g, RPD = 3.66, and RPIQ = 3.22) and MC (*R*^2^*p* = 0.80, RMSEP = 1.91%, RPD = 2.31, RPIQ = 2.62). Overall the study concluded that SF approach with SOPLS attained better performance over the traditional model developed with the single sensor spectra, hence, SF is recommended as the best SF method for improving the prediction accuracy of studied soil attributes. Moreover, the multi-sensor spectra fusion approach is not limited for only MIR and XRF data but in general can be extended for complementary information fusion in order to improve the model performance in precision agriculture (PA) applications.

## 1. Introduction

Assessment of the within field spatial variability in soil fertility is important in precision agriculture (PA) for performing several variable rate operations such as tillage, fertilization, irrigation and seeding [1]. Soil fertility attributes such as, pH, organic carbon (OC), phosphorus (P), potassium (K), magnesium (Mg), calcium (Ca) and moisture content (MC) are considered the most significant indicators associated with soil quality and crop productivity [2,3]. Therefore, accurate measurement of these attributes is important to make better farming decision in PA. In this effort, traditional laboratory-based methods are often used for the measurement of soil attributes while, these methods require specialized equipment, which is labor intensive, costly, time-consuming, and destructive in nature, prohibiting their use in real-time quality measurement. In contrast spectroscopy has been considered as the most promising alternative technique to the traditional method for measurement of soil indicators [4,5]. Spectroscopy is simple, cost-effective, non-destructive and a rapid analytical technique, need minimum or no sample preparation that can be used in online or offline modes for measurement of soil quality indicators [3].

In soil science, mid-infrared (MIR) spectroscopy is one of the most important optical techniques available for qualitative and quantitative analysis of soil fertility and quality. The MIR spectral region (400–4000 cm^−1^) is especially important because fundamental vibrations of the molecules, related to key soil fertility attributes take place [6]. MIR is becoming more common, due to its specificity and well-defined absorption bands of principal constituents (e.g., organic carbon, clay minerals and moisture contents) of the soil in this spectral region. Various studies have investigated the potential of MIR spectroscopy for successful determination of soil properties [7,8,9,10,11]. Likewise, another spectroscopic technique, X-ray fluorescence (XRF) spectroscopy provide the characteristic elemental information (for e.g., Mg, P, K, Ca) of the measured soil. The portable XRF technique is non-invasive, cost-effective, non-destructive, and can be applied for both field and laboratory conditions. In this technique soil atoms are irradiated with X-rays and they emit a secondary florescence radiation and most of the elements present from sodium to uranium in the periodic table are detected. Usage of XRF spectroscopy in soil science has been reported by several authors for successful determination of soil elemental composition [4,12,13,14,15]. Most previous reports used each of these optical methods individually for prediction of soil properties.

Although the individual use of MIR and XRF technique have proven their effectiveness to estimate soil fertility attributes with different degree of success, single sensor can barely measure all soil related attributes alone [2]. In addition, data collected with a single sensor sometimes does not hold comprehensive information of the measured sample to allow accurate predictions of target attributes. Therefore, it is essential to explore spectra fusion (SF) approaches that can handle multi-sensor data simultaneously and assess majority of soil fertility attributes accurately and rapidly.

Recently SF approaches played an important role in providing vast and improved amount of information. Reports demonstrated that SF modelling may improve the accuracy of predictions for the spectral analysis [16]. In this context, advance chemometric tools such as multi-block chemometric strategy could be considered for the purpose of analyzing complex data. The multi-block chemometric strategy can be defined as the merging of data blocks from two or more sensors in a single model. It produces a more comprehensive dataset, enhancing data visualization, identifying key variables, improving predictive performance and allowing deeper data interpretation [17]. Thus, it is more effective to extract information by handling all the blocks at the same time, instead of building individual models for each set of data. For soil spectroscopy analysis there are several SF methods for this purpose; the most well-known standard analysis method is multi-block PLS regression. Recently, the multi-block SF method, named Sequential Orthogonalized Partial Least square (SOPLS) became highly popular in the food sector to extract relevant information from multi sensor data to predict chemical composition in various food products [18,19]. The advantage of SOPLS is its ability to process multiple source data simultaneously (including both regression and discrimination). Correlations between each block of predictors and the response(s) are sequentially calculated after orthogonalization with respect to the scores of previous regressions. Therefore, it can easily handle blocks with more than one latent variable in comparison with standard analytical methods [20]. Such modeling combination already showed improvement in the prediction accuracy of non-soil samples [21]. Which may anticipate a great potential of the methodology to improve the prediction accuracy for soil fertility attributes. Although some studies reported SF methods for soil prediction based on Regression kriging (RK) for fusion of Geophysical and hyperspectral data, Principle Component Analysis (PCA), Outer Product Analysis (OPA), Least–square (LS) and Granger–Ramanathan (GR) for fusion of near-infrared (NIR), MIR and XRF data [5,22,23,24,25], to the best of our knowledge, no previous study has reported the use of SOPLS based SF method in soil science. Moreover, to date no study has reported the combined prediction of soil fertility parameters based on SOPLS based SF of MIR and XRF data.

Motivated by the above considerations, the overall study aims to investigate the performance of SF-SOPLS models against the traditional SF-PLS model and individual sensor spectra models [traditional partial least squares (TPLS)] in improvement of the prediction accuracy of MIR and XRF data for key soil fertility properties (i.e., OC, MC, pH, P, K, Ca, Mg).

## 2. Materials and Methods

### 2.1. Study Sites and Soil Sampling

**Figure 1:**
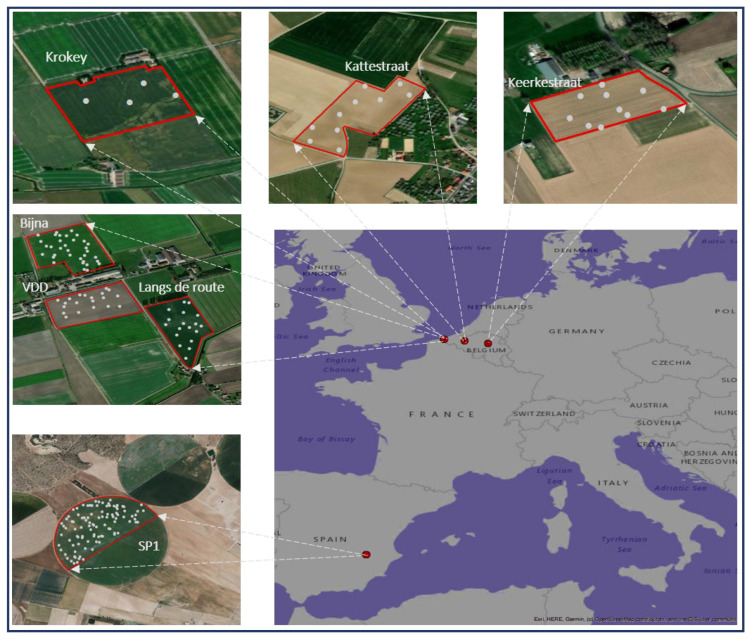
Location of seven experiment fields with sampling points in Belgium and Spain (described in Table 1).

In this study a total of 196 soil samples were randomly collected from agricultural fields at different locations in Belgium and Spain. The samples were taken at 10–20 cm soil depth, with an average spatial sampling rate of 3.25 samples/ha. The fields included are: one field in Spain, designated SP1 (38.776888° N, 1.838478° E), and six fields in Belgium, designated Keerkestraat (50.918051° N, 3.732146° E), Krokey (50.999652° N, 2.548878° E), Kattestraat (50.780363° N, 5.071657° E), VDD Tegen ti hof (51.021233° N, 2.574553° E), Langs de route (51.017723° N, 2.581572° E), and Bijna vrij (51.023043° N, 2.576173° E). The detailed information about the studied fields is provided in Table 1 and sampling area map is shown in Figure 1. Soil samples were brought to the laboratory and were properly cleaned by removing non-soil particles such as plant residues, stones and other debris. The cleaned soil samples were mixed properly by following the standard coning and quartering method [26]. Further, one portion (consisting of 200 g soil/sample) was used for sensor measurement (MIR and XRF measurement), and other portion (consisting of 200 g soil/sample) was used for laboratory chemical analysis of pH, OC, P, K, Mg, Ca and MC determination.

### 2.2. MIR Measurement of Soil Samples

**Figure 2:**
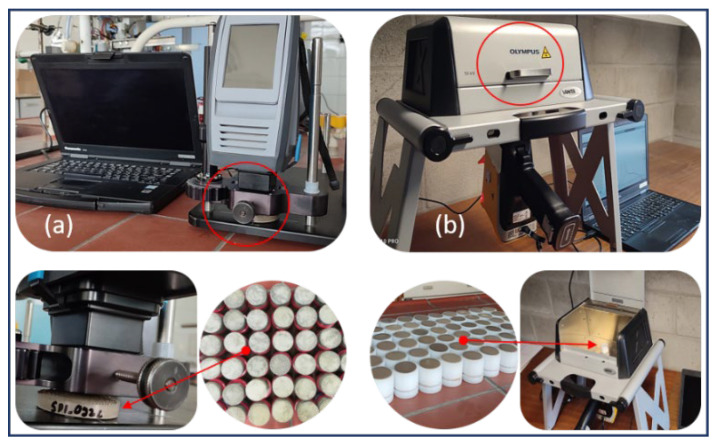
Experiment process for soil measurement. (**a**) mid infrared (MIR) scanning of soil samples, (**b**) X-ray fluorescence (XRF) scanning of soil samples.

Soil samples were first air dried, grinded, and sieved with a 2 mm mesh. For MIR scanning of the processed soil samples, approximately 50 g of each air-dried (at 25 °C for three weeks) and sieved soil sample was placed in a Petri dish (1.0 cm height by 5.5 cm in diameter) and gentle pressure was applied on the surface with a spatula to generate a levelled and smooth surface to ensure the maximum signal-to-noise ratio. A detailed procedure about soil sampling can be found in [8,27]. Three replicates (50 g each) of each sample were prepared following this method. Samples were scanned with the MIR spectrometer (Agilent Technologies, Santa Clara, CA, USA), with a spectral wavenumber range of 4000–650 cm^−1^ at 8 cm^−1^ resolution and 3.73 cm^−1^ sampling interval. Prior to the soil scanning, a background was also obtained (at interval of 30 min) with a silver-plated reference to calibrate the instrument. The spectral data were collected with absorbance mode using the Microlab software V5.0 supplied with the spectrometer and exported in txt format. Average absorbance of the three scans was used for further analysis. Figure 2a shows the experiment process of soil samples with MIR measurement.

### 2.3. X-ray Fluorescence (XRF) Measurement of Soil Samples

About 10 g of each air-dried and sieved soil sample was placed on a 30 mm open-ended XRF cup of 31 mm diameter (n. 1530, Chemplex Industries Inc., Palm City, FL, USA) sealed at the bottom with a 5-µm thick polypropylene film (n. 3520, SPEX, Costa Mesa, CA, USA). A Vanta VMR M-Series handheld XRF scanner (Olympus, Hamburg, Germany), equipped with a Rh X-ray tube (4 W, max. 50 kV, max. 200 µA) and an integrated large-area silicon drift detector (165 eV) was used. Because of safety, the XRF working station (benchtop mode) was used when operating with the XRF device. The samples were put over the measurement window and scanned in triplicate in two operating conditions (15 kV at 30 µA; and 45 kV at 30 µA) by moving the sample cups over the measurement window of the scanner. The three records were then averaged to obtain final elemental concentration for each sample. The spectra were normalized by the detector live time and exported in counts of photons per second (cps). A detailed description about XRF spectra normalization can be found in [5]. Figure 2b shows the experimental process of soil samples with XRF measurement.

### 2.4. Laboratory Measured Soil Properties

The other set of samples was sieved (<2 mm), homogenized and sent to the soil service Belgium (BDB) for reference laboratory chemical analysis of pH, OC, P, K, Mg and Ca. Table 2 shows the descriptive statistics of measured soil attributes. Soil pH was measured in the supernatant, after shaking and equilibration for 2 h in 1 mol/potassium chloride solution (KCI), using 1:2.5 soil: solution ratio. Soil OC was determined using the dry combustion following Dumas principle (ISO 10694; CMA/2/II/A.7; BOC) [7]. For the determination of the OC content, total inorganic carbon (TIC) compounds were in advance removed by treating the soil sample with hydrochloric acid. The ammonium lactate extracted P, K, Mg and Ca were analyzed using inductively coupled plasma atomic emission spectroscopy (ISO 11885; CMA 2/I/B1) [3]. MC was analyzed using air-drying method [7].

### 2.5. Spectra Pre-Treatment

The measured spectral data from MIR and XRF were imported in MATLAB software for successive data processing. The spectral data contain random noise, and spectral variation generated by the sensor therefore, different pre-treatment steps were considered before subjecting the data to multivariate analysis. At first the raw data were smoothed by a moving average method of size 5, which was successively followed by normalization, multiplicative scatter correction (MSC), standard normal variate (SNV) and Savitzky-Golay (SG) filtering. After several rounds of trials, the best preprocessing steps were used for further analysis. The maximum normalization method was used, as it fits the spectral data within unity so that all values would range from 0 to 1 [28]. The scatter correction by the MSC technique is widely used to correct the additive scatter effect from data [29]. It fits a regression line to each sample spectrum by averaging the spectral values obtained at each wavelength using the least squares method. SNV transformation is a normalization method that addresses the slope variation of sample spectra by centering and scaling the individual spectra of the sample. In addition, the SG filtering technique is used to smoothen spectra by removing baseline variations and overlapping peaks [28]. For XRF data an extra preprocessing steps including baseline correction and Compton normalization were also implemented. A more detailed description about Compton normalization can be found in [5]. With each preprocessing technique, prediction models (SF-PLS, SF-SOPLS and SF-VIP-SOPLS) were developed, whose prediction performance was evaluated by means of coefficient of determination (*R*^2^), residual prediction deviation (RPD), ratio of performance to interquartile distance (RPIQ) and root mean square error (RMSE) values. The preprocessing technique showing the highest *R*^2^, RPD and RPIQ and lowest RMSE values was considered to lead to the best performing model. Table 3 shows the best preprocessing steps used for the correction of the soil MIR and XRF data.

### 2.6. Data Preparation

Prior to the creation of the prediction models, the preprocessed spectral dataset (X and Y matrix data) from both sensors was divided into training and test set by Kenard-Stone (KS) algorithm, which divides the samples in a uniform manner by calculating the Euclidean distances between the X variables [30]. By using the KS method 80% of the data (*n* = 156) were used to build the training dataset, while 20% of the data (*n* = 40) were used as a test dataset. Further, the model was constructed using the training set, while the test set was retained for testing the model performance. In this study two kinds of chemometric models were adopted for the analysis of soil data that includes, TPLS model for single sensor spectral data modeling, and spectral-fusion models by concatenated MIR and XRF spectral data. In this study all kinds of data preprocessing, data partition, and data modeling were accomplished in MATLAB software (version 2020b; MathWorks, Natick, MA, USA).

### 2.7. Single Sensor Modeling

For the single sensor modeling, TPLS regression analysis was carried out individually for MIR and XRF data to establish models to predict the soil properties under consideration. In this paper, the individual sensor models were designated as MIR-TPLS and XRF-TPLS.

#### 2.7.1. Traditional Partial Least Squares (TPLS)

TPLS is the most widely used chemometric tool for processing large amounts of spectroscopic data. It is used to solve multicollinearity problems that arise when two or more predictor variables are highly correlated. It can be used for both regression and classification (e.g., partial least squares discriminant analysis) purposes. The analysis determines the linear relationship between X (independent variable) and Y (dependent variables) and predicts the behavior of the Y. In PLS regression, data decomposed into orthogonal structures called latent variables (LVs). The LVs describe the maximum covariance between the spectral data and the response variables [29]. The general model of PLS is defined as follows:

(1) $$X={\mathrm{TP}}^{T}+E$$

(2) $$Y={\mathrm{UQ}}^{T}+F$$

where Y is the matrix of dependent variables corresponding to the measured sample values from the reference soil analysis methods, and X is the n × p matrix of independent variables corresponding to the spectral variables for each measurement. The matrix X decomposes into the score matrix T, loading matrix P, and error matrix E. The matrix Y decomposes into the score matrix U, loading matrix Q, and error matrix F. In addition, scores T and U are connected by the inner linear relationship. In this work, MIR-TPLS and XRF-TPLS models for all studied soil attributes were developed using the training set.

### 2.8. Spectra Fusion (SF) Modeling

Before modelling, the MIR and XRF spectra and the laboratory measured soil attributes were concatenated in one matrix. The resulting matrixes for each soil attributes were subjected to three kinds of regression analysis including SF-PLS, SF-SOPLS and SF-VIP-SOPLS, whose detailed description is provided in the following sections.

#### 2.8.1. SF-PLS

The concatenated spectra of both sensors were used to build a PLS calibration models for the studied soil attributes. Details description of PLS regression is provided in Section 2.7.1.

#### 2.8.2. SF-SOPLS

The second multiblock data-fusion method namely, SOPLS was adopted to solve the regression problem for soil dataset. SOPLS belongs to the family of multi-block PLS-method. It allows easy handling of large collinear variables (blocks) and it is not affected by variances of the blocks thus, particularly suitable for spectroscopic data [31,32]. SOPLS approach uses a matrix orthogonalization operation to extract complementary information sequentially from each data blocks or sensors (in this study MIR and XRF sensors). In this process, the extraction of information is sequential, so that blocks of data are incorporated one at a time, and their incremental contribution is then assessed. In this study the first block was built with MIR data as it is more informative, easy to use and nonhazardous to the samples. While the second block was built with XRF data due to its data complexity and non-ionizing characteristics. For detailed information of SO-PLS method, the readers are referred to the following references [18,21]. A standard linear model of SOPLS algorithm is given as:

(3) $$Y=X_{1}B_{1}\;+\;X_{2}C_{2}+E\;$$

where *Y* is the response matrix; *X*_1_ and *X*_2_ are the data blocks; *B*_1_ and *C*_2_ represent the regression coefficients respectively and *E* is a residual matrix. SOPLS model involves the following steps:

Step 1: *Y* response is fitted to *X*_1_ by PLS regression

Step 2: *X*_2_ is orthogonalized with respect to *X*_1_-scores of the PLS regression extracted in Step (1), obtaining $X{\;}_{2}^{orth}$

Step 3: $X{\;}_{2}^{orth}$ is used to predict the *Y*-residuals obtained from Step (1)

Step 4: The final regression model is obtained by summing up the predications of Step (1) and Step (3), and can be expressed as:

(4) $$\hat{Y}\;=\;X_{1}B_{1}\;+\;X{\;}_{2}^{orth}C{\;}_{2}^{orth}\;=\;T_{X1}\;Q{\;}_{X1}^{T}\;+\;T_{X{\;}_{2}^{orth}}\;Q{\;}_{X{\;}_{2}^{orth}}^{T}\;$$

where $\hat{Y}$ indicates the model predictions $B_{1}$ and $C{\;}_{2}^{orth}$ are the regression coefficient matrices, while *T* and *Q* are the *X*-scores and *Y*-loadings, respectively.

#### 2.8.3. SF-VIP-SOPLS

The SF-VIP-SOPLS model is similar to the SF-SOPLS model however the only difference lies in using variable selection step (based on MIR and XRF spectra) before applying SOPLS model. The selected variables are than concatenated to develop a SOPLS model. Therefore, instead of using a full spectrum (variables) the SF-VIP-SOPLS model is developed based on few important variables for prediction. For the selection of important variables, we used variable impotence in projection (VIP) method. VIP calculates the value of each predictor by fitting the PLS model according to the contribution of both dependent and independent variables. The idea behind this measure is to accumulate the importance of each variable j reflected by w from each component [33]. The VIP for *j*-th variable is defined as:

(5) $$VIP_{j}\;=\;\sqrt{\;\frac{p\sum_{a=1}^{A}W_{ja}^{2}\times SSY_{a}}{SSY_{total}}}\;$$

where *p* is the number of variables, $W_{ja}$ is the weight value for the *j*-th variable of component a, $SSY_{a}$ is the sum of squares of the explained for the ath component, $SSY_{toal}$ is the total sum of square explained for the dependent variable, and A is the total number of components.

The weight value of the PLS model describes the covariance between the dependent and independent variables. Thus, the VIP value, which is based on the PLS weight, reflects important information about the variables contributing to the description of the dependent variables from the independent variables. Therefore, VIP was implemented to select the effective wavelengths that can contribute the most to predicting the soil attributes under consideration. The average of the squared values of the VIP is equal to 1 and generally used as the criterion for important variable selection. Therefore, in this study we implemented a threshold value of 1 for selection of important variables. Eventually, the SOPLS model was developed with wavebands that indicated VIP values above the threshold level.

### 2.9. Methods for Model Evaluation

Moreover, the choice of optimal number of latent variables for single sensor models and spectra fusion models is important, and usually optimized during the cross-validation (CV), by adopting the LV that result in the lowest root mean square error (RMSE) value (Equation (1)). The prediction efficiencies of the developed models were assessed using *R*^2^, RMSE, RPD, and RPIQ. Generally, a satisfactory regression model should have high *R*^2^, RPD, and RPIQ values and low RMSE values. Equations (6)–(9) show the mathematical expression of RMSE, R^2^, RPIQ and RPD, respectively:

(6) $$\mathrm{RMSE}=\sqrt{\;\frac{1}{n}\sum_{i=1}^{n}{\left(y_{i}-{\hat{y}}_{i}\right)}^{2}}$$

(7) $$R^{2}=\frac{\sum_{i=1}^{n}{\left(y_{i}-{\hat{y}}_{i}\right)}^{2}}{\sum_{i=1}^{n}{\left(y_{i}-{\bar{y}}_{i}\right)}^{2}}$$

(8) $$\mathrm{RPIQ}=\frac{Q_{3}-{\;Q}_{1}}{\mathrm{RMSE}}$$

(9) $$\mathrm{RPD}=\frac{\mathrm{SD}}{\mathrm{RMSE}}$$

where ${\hat{y}}_{i}$ and $y_{i}$ are the predicted and measured API concentration values, respectively, n is the number of observations in the prediction set, and ${\bar{y}}_{i}$ is the mean of measured values, $Q_{1}$ and $Q_{3}$ are the upper bound of first and third quartiles of the measurements, SD is the standard deviation.

## 3. Results and Discussion

### 3.1. Spectral Characteristics of Soil Samples

**Figure 3:**
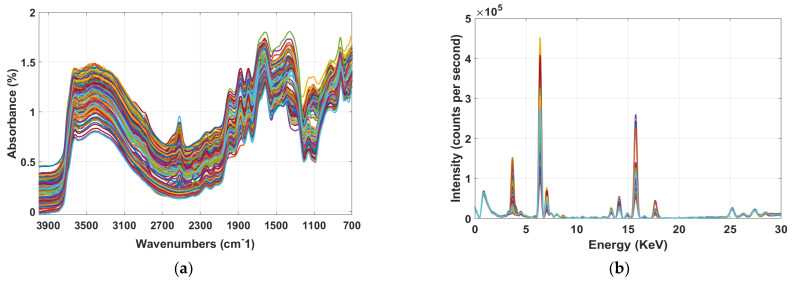
The mid-infrared (MIR) (**a**) and X-ray florescence (XRF) spectra (**b**) of samples used during the study.

Figure 3a,b displays the raw spectra of soil samples for MIR and XRF. By looking at the MIR spectral profile (Figure 3a), it is apparent that the spectra consist of several absorption bands related to the studied soil fertility attributes. These absorption bands in MIR region are much stronger than the those in visible near-infrared (Vis-NIR) region for soil constituents, where overtones and combinations of the fundamental molecular vibrations in the MIR can be observed. For instance, in MIR functional region the spectral signatures between 3800–3000 cm^−1^ are related to the stretching vibration of H-O bonds. The bands at 3000–2820, 1730 and 1873 cm^−1^ are related to the C-H and C=O bonds, whereas the region between 1632–1530 cm^−1^ corresponds to the stretching and banding vibration of both O-H and C-H groups. In addition, the MIR fingerprint regions around 1409 and 1157 cm^−1^ are associated to the C-H and C-O bending vibration [34]. It is worth mentioning that the aforementioned vibrations of soil are aroused due to the primary properties (e.g., mainly OC, and MC), which have a direct MIR spectral response whereas, the secondary properties (e.g., pH, P, K, Mg, Ca) have an indirect correlation with the primary properties [3,26]. Based on this hypothesis, the MIR bands corresponding with primary properties can also be used to detect the secondary properties of the soil properties through covariation.

In XRF plot (Figure 3b) the heavy elements (atomic number > 16) such as Ca showed strong peak between 3–5 KeV [35] while, emission lines for lighter elements (atomic number < 16) such as P and Mg are too weak to be noticed in this region. Since a high resolution (SDD) detector (165 eV) was used in this study, it is odd to observe the overlap between the K-Ka (3314 eV) and Ca-Ka (3692 eV) emissions. However, this overlap is perhaps due to the very low intensity in that region, and when zoomed in a clear separation can be observed (not shown in this work), which is in line with findings by Tavares et al. (2020) [36]. In addition, the spectral region between 5–8 KeV is associated with Fe [37]. The XRF spectra also showed peaks between 13–17 KeV and 25–28 KeV, however for this particular study these regions are insignificant as there is no spectral characteristics related with the studied soil properties. Although it is expected that Rh K lines scattering peaks to appear around 20 keV, they are not seen in this work due to very low intensity in this region. A similar spectral pattern was observed also by Tavares et al. (2020) [36]. The peak at 2.5 keV is the noise form the detector, which was removed during spectral preprocessing and model development.

### 3.2. Results of Single Sensor Modeling Based on PLS (MIR-TPLS and XRF-TPLS)

At first, prediction models were developed and validated individually for each spectroscopic block (MIR and XRF spectra), to investigate how efficient any of the two spectral technique could be for allowing accurate prediction of the studied soil properties. Results of the prediction accuracy of MIR-TPLS and XRF-TPLS models for the training (for cross-validation) and the test sets are summarized in Table 4. In all the cases, MIR-TPLS model prediction accuracy was higher than the XRF-TPLS model in terms of *R*^2^*p*, RPD and RPIQ for all studied soil attributes. Despite this, comparable results of the two techniques were observed for pH and P prediction. This perhaps due to the fact that MIR region holds plenty functional information and fingerprint regions associated with soil attributes investigated, while XRF is well known to be successful for the detection of the total content of soil nutrients (e.g., Mg, Ca) [37]. Since in this study we consider the extractable contents of the nutrients, XRF underperformed MIR. For pH, P and Ca prediction, both MIR-TPLS and XRF-TPLS models exhibited the highest *R*^2^*p* (ranged between 0.71 and 0.89) with acceptable RPD, RPIQ, and RMSEP values. For Mg prediction, the MIR-TPLS model yielded better accuracy (*R*^2^*p* = 0.74, RPD = 1.98, RPIQ = 1.76) than that of XRF-TPLS (*R*^2^*p* = 0.59, RPD = 1.50, RPIQ = 0.99). MIR-TPLS prediction performance for MC (*R*^2^*p* = 0.71, RPD = 1.85, RPIQ = 2.26) was also better than XRF-TPLS (*R*^2^*p* = 0.67, RPD = 1.67, RPIQ = 1.57). However, XRF-TPLS completely failed to predict OC and K components, while MIR-TPLS provided moderate prediction result to OC and MC (*R*^2^*p* = 0.63, RPD = 1.63, RPIQ = 1.66), which was surprising as OC has direct spectral response due to fundamental vibrations in MIR [38].

### 3.3. Results of Fusion Model Based on SF-PLS and SF-SOPLS

**Figure 4:**
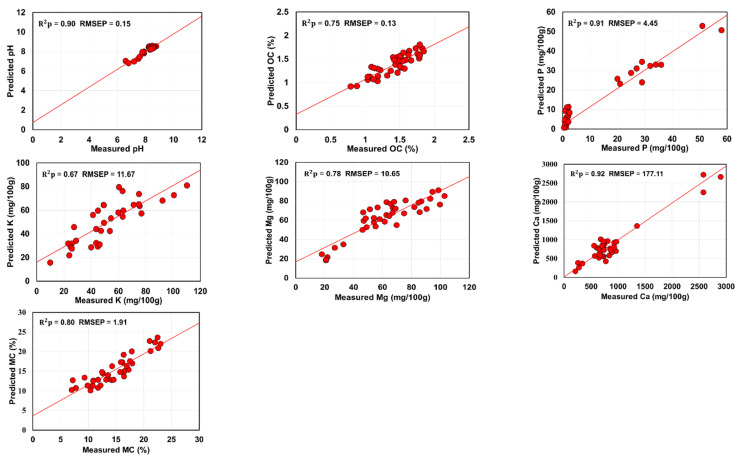
Predicted vs. measured scatter plots of best spectra fusion method (SF-SOPLS) for predicting seven soil attributes in test-set. Units of RMSEP are the same as units of respective soil properties.

To improve the prediction performance, spectral fusion models were built by concatenating spectra of MIR and XRF sensors. The performance of SF-SOPLS models was presented in Table 4. It can be observed from Table 4 that SF-SOPLS model prediction accuracy was higher compared with both individual models (MIR-PLS and MIR-XRF) and SF models (SF-PLS and SF-VIP-SOPLS). More specifically, the model developed with SF-SOPLS greatly improved the prediction of all soil attributes in the test sets (Figure 4 and Table 4). Moreover, the SF-SOPLS accuracy was also higher than the results reported by the earlier researchers (based on the data fusion for Vis-NIR and XRF data) for soil quality prediction [22,39]. The higher accuracy might be due to the more efficient data fusion steps involved during SOPLS model in the current work to improve the prediction. Apart from the advantage of SOPLS model, the MIR region (4000–650 cm^−1^) also contributes in improvement of prediction accuracy as this region provided more detailed information (based on fundamental vibrational bands related to the functional groups of the soil) than the Vis-NIR region used by the earlier researchers.

Among all the studied soil properties, SF-PLS and SF-SOPLS models for pH, P and Ca generally had the highest *R*^2^*p*, RPD, RPIQ and lowest RMSEP values. For soil pH prediction, the spectra fusion models (SP1 and SP2) exhibited the highest *R*^2^*p* of 0.88–0.90, RPD of 2.95–3.30, and RPIQ of 2.76–3.59, and lowest RMSEP of 0.15–0.17. For soil P prediction, the second highest *R*^2^*p* of 0.82–0.91, RPD of 2.28–3.53, and RPIQ of 2.04–4.90 and the second lowest RMSEP of 4.45–6.81 mg/100 g were observed. For Ca prediction, the high accuracies were recorded (*R*^2^*p* of 0.73–0.92, RPD of 1.89–3.66, RPIQ of 2.34–3.22 and RMSEP of 177.11–419.32 mg/100g), but not as good as those of pH and P. The prediction of Mg and MC contents was in the moderate category. For Mg prediction *R*^2^*p* of 0.61–0.78, RPD of 1.59–2.17, RPIQ of 1.48–2.13 and RMSEP of 10.65–13.78 mg/100 g were calculated, whereas for soil MC prediction *R*^2^*p* of 0.66–0.80, RPD of 1.70–2.31, RPIQ of 2.09–2.62 and RMSEP of 1.91–2.52% were recorded. However, the SF-PLS model performance was limited for soil K prediction, and only SF-SOPLS model was able to provide maximum values of *R*^2^*p* of 0.67, RPD of 1.77, RPIQ of 2.13 and RMSEP of 11.67 mg/100 g. A similar case was also observed for soil OC prediction, for which SF-PLS model completely failed, while SF-SOPLS model provided good prediction results (*R*^2^*p* = 0.75, RPD = 2.02, RPIQ = 2.47). The prediction accuracy of SF-SOPLS for OC outperformed those obtained by the corresponding individual models. Figure 4 depicts the SF-SOPLS based scatterplots between measured vs predicted soil attributes based on the *R*^2^*p* and RMSEP values listed in Table 4. The slopes for the pH, P and Ca using the test dataset are very well distributed along the 1:1 linear line indicating a best validation, the slopes for the OC, Mg and MC showed moderate distribution, whereas the slops for the K with the test dataset are slightly far from the 1:1 linear line indicating under-estimation of soil property [34].

### 3.4. Results of Fusion Model Based on SF-VIP-SOPLS

**Figure 5:**
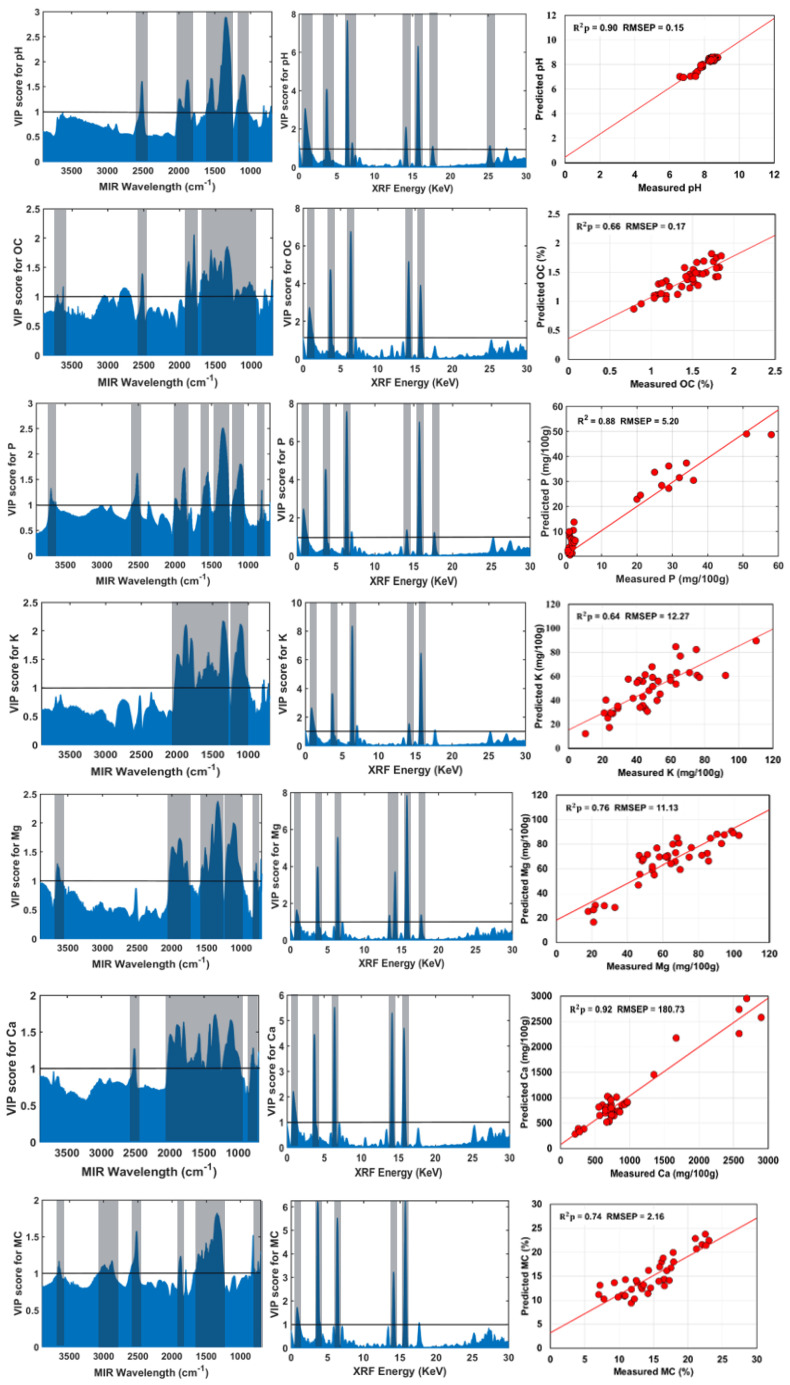
Variable impotence in projection (VIP) score plots for pH, organic carbon (OC), phosphorus (P), potassium (K), magnesium (Mg), calcium (Ca) and moisture content (MC) with corresponding scatter plots of measured against predicted values using the SF-VIP-SOPLS model developed based on selected variables. Units of root mean square error of prediction (RMSEP) are the same as units of respective soil properties. In VIP score plots the solid horizontal lines indicate the threshold values (value = 1) used for variable selection, and the vertical highlighted region is the significant range selected for prediction.

Figure 5 displays the VIP score plots of soil attributes used for variable selection step (during SF-VIP-SOPLS model development), and scatter plots of measured against predicted soil attributes using the SF-VIP-SOPLS model using the selected variables as input to PLS. The prediction results of the SF-VIP-SOPLS models are better than those of the SF-PLS models, although only few numbers of variables were used. While SF-PLS and SF-SOPLS models used the entire spectral variables of 2048, the SF-VIP-SOPLS model uses only 387–524 variables (from MIR and XRF data), providing comparable prediction results to those by SF-SOPLS. The SF-SOPLS model slightly outperformed the corresponding models of SF-VIP-SOPLS (Table 4). Similar to the SF-SOPLS model, once again more accurate results were obtained by the SF-VIP-SOPLS model for pH (*R*^2^*p* = 0.90, RPD = 3.22), P (*R*^2^*p* = 0.88, RPD = 2.99), and Ca (*R*^2^*p* = 0.92, RPD = 3.60) prediction, compared to the individual sensor models. The performance of SF-VIP-SOPLS was also found satisfactory for Mg (*R*^2^*p* = 0.76, RPD = 2.07) and MC (*R*^2^*p* = 0.74, RPD = 2.07) prediction. On the other hand, the model accuracy was dropped for prediction of OC and K contents (*R*^2^*p* < 0.70). However, it is worth mentioning that with only 387–524 selected variables the SF-VIP-SOPLS model was capable to provide satisfactory prediction, that was not possible with SF-PLS, MIR-PLS and XRF-PLS models. This is due to fact that most of picked variables (by VIP method) in SF-VIP-SOPLS model comprise chemical and elemental information that contribute to the correct model prediction.

In the VIP score plot for pH (Figure 5), 406 variables in the range from 2580–2306, 2137–2098, 1709–1689 and 1556–1400 cm^−1^ related to the O-H stretching vibration, O-H deformation vibration and C=O groups, respectively [40] are found to be significant. For OC prediction, 524 variables in range of 1220–1530, 1700–1880 and 3600–3700 cm^−1^ related to the C-H, C-O and O-H groups, respectively are found to be significant [7,34]. For P prediction, 387 significant variables were observed in the spectral bands of 800–900, 1100–1200, 1300–1400, 1500–1600, 1800–2000 and 3600 cm^−1^ For K prediction, 453 variables are significant in the range of 1100–1200 and 1400–2000 cm^−1^. For Mg prediction, 449 variables are found significant in the range of 800–900, 1100–1200, 1300–1500, 1800–2000 and 3600 cm^−1^. For Ca prediction, 503 variables are significant in the range of 800–900, 1100–1200, 1400–2000 and 2300–2500 cm^−1^. Finally, for the MC, 503 variables are found to be significant in the range of 800–900, 1300–1700, 1900, 2580–2306, and 3600 cm^−1^. The results suggested that SF-VIP-SOPLS models are preferable, as they reduce the data dimensionality by selecting the important variables while preserving the relevant information related to studied soil fertility parameters. Comparing among the five different models, the XRF-PLS and SF-PLS is the lowest performing models, whereas SF-SOPLS followed by SF-VIP-SOPLS are the best performing models for the prediction of all soil parameters.

## 4. Conclusions

This study adopted a SOPLS based spectra fusion (SF) technique of MIR and XRF data for the first time in soil analysis for the prediction of soil pH, OC, P, K, Mg, Ca and MC. Both MIR and XRF data were concatenated in the spectra fusion analysis, and the results of the output models were compared with corresponding models developed with individual (MIR or XRF) spectra. Results achieved in this work support the following conclusions:

- The individual MIR-PLS model exhibited a better prediction accuracy than the individual XRF-PLS model.
- For SF-PLS model no improvement in prediction accuracy was observed for all studied soil properties.
- The SF-SOPLS model showed the highest improvement in the prediction accuracy, compared with other models for all studied soil properties, with the largest improvement obtained for pH, P, and Ca prediction.
- The SF-VIP-SOPLS models’ prediction accuracy was higher than those of the MIR-PLS, XRF-PLS and SF-PLS models, while slightly lower than the corresponding SF-SOPLS models.

While SF-SOPLS models outperformed compared with the traditional PLS SF and the individual models for improving the prediction performance of all studied soil attributes. The VIP based SOPLS SF models can be recommended as the best modelling option to be used. The advantage of VIP based SOPLS models (SF-VIP-SOPLS) is that the resulted models can discard the redundant variables from the data set, hence, minimize the risk of overfitting, noise, and nonlinearities in the model. Ultimately, since smaller number of variables are used, this makes these models fast and easy computations while maintaining the model accuracy. However, further work can be considered to test SOPLS model by spectra fusion of other types of sensor data (e.g., vis-NIR, MIR and XRF) to improve prediction of soil parameters. In addition, the results presented in this paper confirms the potential of SF, especially the SOPLS and VIP model, in improvement of the prediction performance of the studied soil properties. This improvement can be applied in precision agriculture for accurate estimations of key fertility attributes necessary for making accurate and advanced decisions.

## Figures and Tables

**Table 1:** Characteristics of the study fields in Spain and Belgium.

Field	Period	Area (ha)	Crop Type	N	Soil Texture	Average MC (%)	Average OC (%)
SP1, Spain	2019	50	Opium, Garlic	100	Clay loam	13.18	1.48
Keerkestraat, Belgium	2020	1.2	Maize	10	Loam	21.63	1.26
Krokey, Belgium	2020	13	Oil seed rape	4	Loam	19.31	1.66
Kattestraat, Belgium	2020	5	Potatoes	9	Loam	18.00	1.27
VDD Tegen ti hof, Belgium	2020	5	Potatoes	20	Loam	16.61	1.50
Langs de route, Belgium	2020	6	Potatoes	18	Polder	17.78	1.12
Bijna vrij, Belgium	2020	7	Sprout	35	Polder	22.43	1.08

N = number of samples; MC = moisture content; OC = organic carbon.

**Table 2:** Descriptive statistics of laboratory measured soil attributes for selected sample sets used for building training and test sets.

Soil Indicators	N	Sample Set	Range	Mean ± SD
pH	156	Training set	6.50–8.65	8.02 ± 0.50
	40	Test set	6.60–8.76	8.19 ± 0.51
OC (%)	156	Training set	0.73–2.47	1.34 ± 0.30
	40	Test set	0.79–1.84	1.43 ± 0.28
P (mg/100 g)	156	Training set	0.33–69	19.21 ± 18.77
	40	Test set	0.51–58	10.31 ± 15.59
K (mg/100 g)	156	Training set	9.00–122.91	41.56 ± 22.46
	40	Test set	10.00–110.28	48.81 ± 20.72
Mg (mg/100 g)	156	Training set	17.00–175.29	62.25 ± 23.78
	40	Test set	18.00–102.91	62.48 ± 23.12
Ca (mg/100 g)	156	Training set	196.00–3880	1380 ± 956.93
	40	Test set	212.00–2900	929.23 ± 652.40
MC (%)	156	Training set	9.01–26.01	16.75 ± 4.33
	40	Test set	7.02–23.05	14.83 ± 4.31

N = number of samples; OC = organic carbon; P = Phosphorous; K = potassium; Mg = Magnesium; Ca = Calcium; MC = Moisture content; SD = standard deviation.

**Table 3:** Best preprocessing steps considered for correction of mid infrared (MIR) and X-ray fluorescence (XRF) spectral data.

Data	Spectral Pretreatment	Soil Quality Indicators
MIR	Moving average → Normalization	pH, OC, Mg, MC
MIR	Moving average → SNV	P
MIR	Moving average	K
MIR	Moving average → MSC	Ca
XRF	Baseline correction → Compton normalization → Moving average → Normalization	pH, OC, Mg, MC
XRF	Baseline correction → Compton normalization → Moving average → SNV	P
XRF	Baseline correction → Compton normalization → Moving average	K
XRF	Baseline correction → Compton normalization → Moving average → MSC	Ca

MIR = mid-infrared; XRF = X-ray fluorescence; SNV = Standard normal variate; MSC: Multiplicative scatter correction; OC = organic carbon; P = Phosphorous; K = potassium; Mg = Magnesium; Ca = Calcium; MC = Moisture content; SD = standard deviation.

**Table 4:** Prediction results of soil pH, organic carbon (OC), phosphorous (P), potassium (K), magnesium (Mg), calcium (Ca), moisture content (MC) using traditional PLS model (TPLS), and spectra-fusion (SF-PLS, SF-VIP-SOPLS and SF-SOPLS).

Soil Indicators	Model Type	Training Set			Test Set				
		*R* ^2^ *cv*	RMSEC	RPD	*R* ^2^ *p*	RMSEP	RPD	RPIQ	Variables
pH	MIR-TPLS	0.90	0.15	3.19	0.89	0.16	3.03	3.51	908
	XRF-TPLS	0.89	0.16	3.07	0.88	0.17	2.95	2.78	2048
	SF-PLS	0.89	0.16	2.66	0.88	0.17	2.95	2.76	2948
	SF-SOPLS	0.94	0.11	4.14	0.90	0.15	3.30	3.59	2948
	SF-VIP-SOPLS	0.91	0.14	3.49	0.90	0.15	3.22	3.54	406
OC (%)	MIR-TPLS	0.76	0.14	2.05	0.63	0.17	1.63	1.66	900
	XRF-TPLS	0.59	0.19	1.57	0.30	0.24	1.18	1.01	2048
	SF-PLS	0.56	0.19	1.50	0.35	0.22	1.24	1.11	2948
	SF-SOPLS	0.75	0.13	2.05	0.75	0.13	2.02	2.47	2948
	SF-VIP-SOPLS	0.78	0.13	2.17	0.66	0.17	1.70	2.09	524
P (mg/100 g)	MIR-TPLS	0.87	6.74	2.78	0.84	7.73	2.45	2.69	900
	XRF-TPLS	0.85	7.04	2.66	0.83	6.36	2.45	2.34	2048
	SF-PLS	0.90	5.90	3.17	0.82	6.81	2.28	2.04	2948
	SF-SOPLS	0.95	4.11	4.56	0.91	4.45	3.53	4.90	2948
	SF-VIP-SOPLS	0.92	5.27	3.56	0.88	5.20	2.99	2.90	387
K (mg/100 g)	MIR-TPLS	0.71	12.69	1.86	0.65	14.12	1.70	1.90	900
	XRF-TPLS	0.66	13.74	1.72	0.48	15.03	1.37	1.68	2048
	SF-PLS	0.67	12.31	1.74	0.48	14.90	1.39	1.56	2948
	SF-SOPLS	0.72	11.82	1.78	0.67	11.67	1.77	2.13	2948
	SF-VIP-SOPLS	0.76	10.51	2.04	0.64	12.27	1.68	1.67	453
Mg (mg/100 g)	MIR-TPLS	0.77	11.39	2.08	0.74	11.64	1.98	1.76	900
	XRF-TPLS	0.65	13.94	1.70	0.59	15.33	1.50	0.99	2048
	SF-PLS	0.78	10.18	2.16	0.61	13.78	1.59	1.48	2948
	SF-SOPLS	0.80	9.54	2.26	0.78	10.65	2.17	2.13	2948
	SF-VIP-SOPLS	0.79	9.93	2.21	0.76	11.13	2.07	1.87	449
Ca (mg/100 g)	MIR-TPLS	0.91	274.46	3.45	0.85	261.87	2.49	2.70	900
	XRF-TPLS	0.84	372.97	2.54	0.71	466.43	1.81	2.24	2048
	SF-PLS	0.87	331.82	2.85	0.73	419.32	1.89	2.34	2948
	SF-SOPLS	0.96	176.73	5.36	0.92	177.11	3.66	3.22	2948
	SF-VIP-SOPLS	0.96	185.57	5.11	0.92	180.73	3.60	3.20	503
MC (%)	MIR-TPLS	0.81	1.84	2.34	0.71	2.32	1.85	2.26	900
	XRF-TPLS	0.76	2.09	2.07	0.64	2.57	1.67	1.57	2048
	SF-PLS	0.77	2.07	2.08	0.66	2.52	1.70	2.09	2948
	SF-SOPLS	0.85	1.75	2.47	0.80	1.91	2.31	2.62	2948
	SF-VIP-SOPLS	0.86	1.59	2.71	0.74	2.16	2.01	2.49	466

*R*^2^*cv* and *R*^2^*p* = coefficient of determination for cross-validation and prediction; RMSEC and RMSEP = root mean square error of cross-validation and prediction; RPD = Residual prediction deviation; RPIQ = ratio of performance to interquartile distance; MIR-TPLS = mid-infrared-traditional partial least square; XRF-TPLS = X-ray fluorescence-traditional partial least square; SF-PLS = spectra fusion based on partial least square; SF-SOPLS = spectra fusion based on sequential orthogonalized partial least squares; SF-VIP-SOPLS = spectra fusion based on sequential orthogonalized partial least squares with variable importance in projection; OC = organic carbon; P = Phosphorous; K = Potassium; Mg = Magnesium; Ca = Calcium; MC = Moisture content.

## Data Availability

Not applicable.
